# Social Networking App Use Among Primary Health Care Professionals: Web-Based Cross-Sectional Survey

**DOI:** 10.2196/11147

**Published:** 2018-12-21

**Authors:** Francesc X Marin-Gomez, Francesc Garcia Cuyas, Ramon Reig-Bolano, Jacobo Mendioroz, Pere Roura-Poch, Margalida Pico-Nicolau, Josep Vidal-Alaball

**Affiliations:** 1 Servei d'Atenció Primària d'Osona Gerència Territorial de la Catalunya Central Institut Català de la Salut Vic Spain; 2 Unitat de Suport a la Recerca Catalunya Central Institut Universitari d'Investigació en Atenció Primària Jordi Gol Sant Fruitós de Bages Spain; 3 Health Promotion in Rural Areas Research Group Institut Català de la Salut Sant Fruitós de Bages Spain; 4 Digital Care Research Group Centre for Health and Social Care Research Universitat de Vic–Universitat Central de Catalunya Vic Spain; 5 Department of Information and Communications Technology in Health Universitat de Vic–Universitat Central de Catalunya Vic Spain; 6 Department of Engineering Universitat de Vic–Universitat Central de Catalunya Vic Spain; 7 Centre d'Atenció Primària Sant Quirze de Besora Gerència Territorial de la Catalunya Central Institut Català de la Salut Sant Quirze de Besora Spain

**Keywords:** mHealth, social networking, smartphone, attitude, primary health care, telemedicine, cell phone use

## Abstract

**Background:**

Several studies have been conducted to analyze the role social networks play in communication between patients and health professionals. However, there is a shortage of studies in relation to communication among primary health professionals, in a professional context, using the various mobile phone apps available.

**Objective:**

The objective of our study was to explore mobile phone social networking app use among primary health care professionals for work-related purposes, by comparing the most widely used apps in the market.

**Methods:**

We undertook a cross-sectional study using an anonymous Web survey among a convenience sample of 1635 primary health care professionals during August and September 2017.

**Results:**

Of 483 participants in the survey, 474 (98.1%, 95% CI 97.1%-99.4%) were health professionals who commonly accessed social networking sites and 362 (74.9%, 95% CI 71.1%-78.8%) accessed the sites in a work-related context. Of those 362 respondents, 219 (96.7%, 95% CI 94.8%-98.5%) preferred WhatsApp for both personal and professional uses. Of the 362 respondents who used social networking sites in a work-related context, 276 (76.2%, 95% CI 71.9%-80.6%) rated social networking sites as useful or very useful to solve clinical problems, 261 (72.1%, 95% CI 67.5%-76.7%) to improve their professional knowledge, and 254 (70.2%, 95% CI 65.5%-74.9%) to speed up the transmission of clinical information. Most of them (338/362, 94.8%, 95% CI 92.5%-97.0%) used social networking sites for interprofessional communications, and 204 of 362 (56.4%, 95% CI 51.2%-61.5%) used them for pharmacological-related consultations.

**Conclusions:**

Health professionals frequently accessed social networking sites using their mobile phones and often for work-related issues. This trend suggests that social networking sites may be useful tools in primary care settings, but we need to ensure the security of the data transfer process to make sure that social networking sites are used appropriately. Health institutions need to increase information and training activities to ensure the correct use of these tools.

## Introduction

### Background

The introduction and development of mobile technology and the expansion of social networking are changing social relationships and modifying behaviors and attitudes, especially among the younger generations. Mobile phones are not only used as working tools, but often continue to be used for similar purposes at home, thus extending the normal working hours. In 2016, a survey showed that 90.4% of general practitioners owned a mobile phone with 1 to 3 medical-related apps [1]. There are not many studies on the current use of social networking among primary care professionals and even fewer comparing the use of the various apps available in the market.

Social networking sites (SNSs) are Web-based services that allow individuals to construct a public or semipublic profile within a bounded system to share information, ideas, personal messages, and other content in online communities [2]. Their forms of online communication vary greatly depending on their features (eg, photo-sharing or video-sharing capabilities, built-in blogging, and instant messaging technology). Most Web-based SNSs also support mobile interactions. There are considerably more mobile users than personal computer users, but how individuals decide to access SNSs (ie, through personal computers, iPads, tablets, or mobile phones) still depends on their own choice. Mobile phone apps allow for creating, sharing, and exchanging information, images, or videos with other users through a mobile portable format, and probably this is the main reason why the use of apps has grown rapidly among SNSs. In 2016, the number of apps downloaded to connected devices worldwide was 149.3 billion [3], and there were 3196 million active SNSs users, or about 42% of the global population. Of those, 2958 million, about 39% of the world’s population, accessed SNSs through their mobile devices [4]. Apps such as WhatsApp, Facebook, YouTube, Instagram, Twitter, Spotify, Telegram, LinkedIn, or Snapchat have contributed to increase the number of mobile phone users and, in turn, the number of SNS users around the world. A recent report estimated that in Spain more than 15 million people aged between 16 and 55 years were active users of SNS technologies. WhatsApp and Facebook were the favorite SNSs of mobile phone users (76%) and people spent most of their time in WhatsApp. This increasing use of SNSs has been attributed to their being a way to socialize with peers (to chat or send messages) [5].

From a health care perspective, the use of mobile phones by clinicians could improve clinical communication, increase the practice of evidence-based medicine, enable access to information tools at the point of care, and improve education and research [6-9]. Apps designed for health professionals can be used to diagnose diseases, consult data on medications, perform clinical calculations, search scientific evidence, exchange clinical experiences, improve the management of chronic diseases, and conduct health care research [10]. The benefits of mobile technology for health professionals include the ability to make decisions more quickly and more reliably, thus improving the quality of health care and data management [11,12]. Apps to access SNSs stand out in the improvement of accessibility to health information, both as a support tool and for public health surveillance [13-16]. A greater connection with other professionals has been highlighted as one of the main benefits associated with the use of SNS media in the field of health care. Health professionals, from all categories, are using apps as social media for their professional development, to connect with colleagues, and to be up-to-date with the latest medical literature. Health care organizations around the world are taking initiatives to expand mobile health use and to demonstrate its efficiency [17]. The focus areas for future development of these technologies probably will be mobile telehealth and disease surveillance with SNS media and clinical decision support systems using machine learning. During the recent outbreak of Ebola virus in Africa, mobile phones and their apps were used for research, surveillance, and health education and to follow its dissemination [18,19]. It is likely that the use of apps in cases like these will increase in the future due to their potential to improve the health outcomes of patients in various health care settings.

Few studies have been undertaken on primary care professionals’ use of various apps to access SNSs in a professional context. In a survey [20] on the use of mobile phones at work, in which about half the sample of 416 respondents were registered nurses, 58% of these nurses used their mobile phones at work; this use increased to 81% among physicians. The importance of this phenomenon and its foreseeable future impact require additional research on the use made by health care professionals in all types of social networks and devices. Primary care professionals (physicians, nurses, midwives, medical social workers, etc) are usually establishing the first contact with patients, and this type of SNS app, in a portable format, allows for remote support that seems useful and effective. For this reason, it is necessary to evaluate SNSs’ impact and benefits perceived by members of primary care teams [21].

However, there is a growing fear and some controversies in relation to extending the use of social networks in health data communication contexts, which have their origin in the threat to privacy and confidentiality and the risk of misinformation, fake news, and the impersonation of professionals as recently reported in some media stories [22]. The increase in reports of these situations shows that these are risks to be taken into serious consideration [23]. If we add to this the risks associated with storing and transporting images, multimedia files, or text files on these mobile devices that go wherever the user goes and that often connect through low-reliability Wi-Fi networks, security risks rise exponentially. Lack of clarity on the boundaries between personal and professional life, increased risk of liability arising from the use of SNSs for professional purposes, low methodological rigor in studies on the use of social media, and poor accuracy, quality, and reliability of information are creating serious doubts about extending SNS use among health care professions [6-9,11-15].

### Objectives

Considering the need for more studies on SNS use and the growing trend toward the use of social networks to disseminate and discuss knowledge, we chose Bloom’s taxonomy as an evaluative tool [24-26]. Our aim in this study was integrate this taxonomy into our exploration of primary health care professionals’ use of SNSs and their main reasons for using them.

## Methods

### Design

This was a descriptive cross-sectional study to explore, through a Web-based survey, primary health care professionals’ use of various social networking apps. The survey was conducted anonymously from August to September 2017.

### Sample and Settings

The target population for the survey was a convenience sample of 1635 practicing primary health care professionals registered in SISAP (the Catalan acronym for Information Systems for Primary Care Services) [27] who worked in the central region of Catalonia, Spain. Those invited to take part in this study had an account to access electronic health records, had a valid email address, and had previously given consent to be contacted.

We distributed a link to the questionnaire by email. The email invited potential participants to take voluntary part in the questionnaire and explained the aim of the study.

### Web-Based Survey

We used a voluntarily accessed survey developed using the Google Forms tool (Google LLC, Mountain View, CA, USA). Participants had access to the survey through a link sent in a personalized email. It was a closed survey, and no personal identification data were collected, thus protecting the confidentiality of participants. There was no financial incentive for participating in the study.

We carried out a pilot test with a group of 47 health care professionals (similar to the target group) to ensure the clarity of the questions and the validity of the rating scale. We introduced no major changes.

The first page of the survey informed participants about the total number of questions, the approximate response time, and the aim of the study. Participants were encouraged to contact the main investigator if they had any questions requiring clarification (contact details were also on the same page). The questionnaire, consisting of 10 multiple-choice questions, had only 1 conditional question referring to the use of social networks in a professional context. Depending on the response, it allowed access to a second section. The questionnaire was distributed in 3 distinct sections with all questions, except the last one, being mandatory. Some questions allowed free-text content (eg, apps used) and others allowed combined answers (options were “none” and “all”). As the questions were mandatory, incomplete answers were not registered. Only 1 response was allowed for each email sent. We kept no records of the respondents who quit the survey and analyzed only the completed questionnaires. We did not apply any statistical weighting.

### Study Variables

The survey was divided into 3 sections: (1) type of apps used by the health care professional, (2) type of apps used in a professional context, and (3) professional perception of the benefits and impact of the apps on their clinical practice and professional development. For this last part, professionals were asked about the usefulness of using apps, classifying the answers as “not useful,” “of little use,” “useful,” and “very useful” in terms of their benefits and impact. We used 8 distinct categories based on the 2 dimensions of Bloom’s taxonomy (knowledge and cognitive processes), previously used in similar studies [28]: knowledge, clinical reasoning, critical thinking, clinical skills, problem solving, creativity, decision making, and outcome on the patient. An additional closed-ended question asked respondents to indicate whether they used SNSs for work-related purposes and, if they did, they were asked about the main reasons for this use.

The apps we chose to evaluate in the survey were those reported as being the most used in Spain (Facebook, WhatsApp, Twitter, Instagram, and other) [5]. We collected sociodemographic data (age, sex, education level, and work experience) using a demographic form. We determined professional category (physician, nurse, midwife, odontologist, social worker, or other) using a jobs checklist; we also recorded type of work (classified as “academic only,” “clinical only,” “academic and clinical, “ or “other”) and years of work experience. We did not evaluate the qualitative data collected for this study.

### Ethical Considerations

We obtained ethical approval of the study from the University Institute for Primary Care Research Jordi Gol Clinical Research Ethics Committee (P17/174), Barcelona, Spain. The invitational email described the study’s aims and procedures, and security and confidentiality of data. It also informed invitees about their right to decline to participate. The study observed data protection laws in effect at the time it was conducted.

### Statistical Analysis

We made a bivariate comparison using the Pearson chi-square test between the professionals who used the apps in a professional context and those who did not, considering sociodemographic, professional knowledge, and attitude variables.

We performed a multivariate analysis using logistic regression, including the use of SNSs in a professional context as the dependent variable and taking *P*<.05 in the bivariate analysis. We also determined the adjusted odds ratio (adjusted OR). We conducted the analysis using IBM SPSS version 18 (IBM Corporation) and we reported the summary statistics as frequencies and percentages.

## Results

### Participant Characteristics

**Figure 1:**
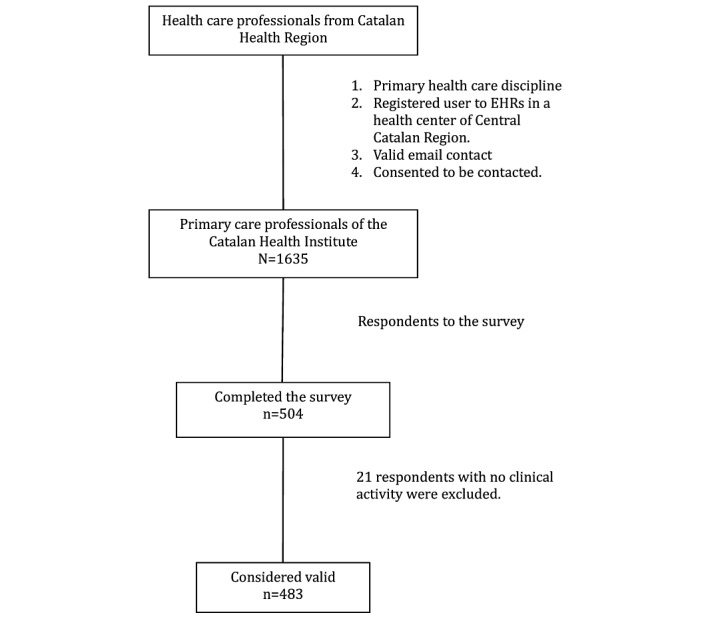
Flow of participants through the study. EHR: electronic health record.

Of the 503 respondents, we included 483 as study participants and excluded 21 who had no clinical activity (ie, academic or research professionals; Figure 1).

The median age of the 483 participants was 45 years (SD 10.44, range 24-65). Most of them (393/483, 81.4%) were women and had a median work experience of 19 years (SD 10.88, range 1-58). In the professional category, 211 of the 483 participants (43.7%) were physicians and 215 (44.5%) were nurses. Of the 483 participants, 385 (75.6%) had a bachelor’s, a graduate, or a diploma degree, and 118 (24.4%) had a master’s or a doctoral degree.

### App Use Analyses

**Figure 2:**
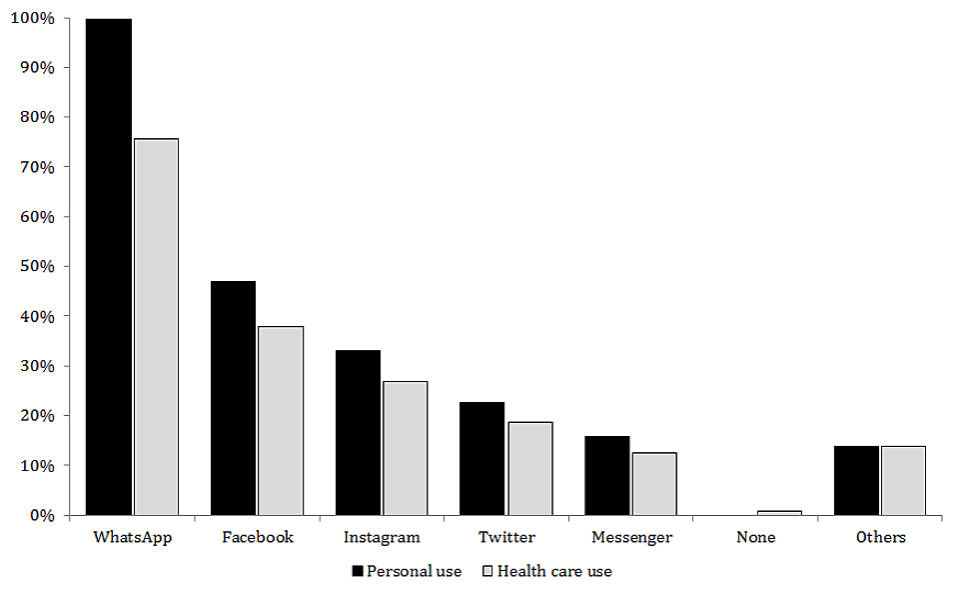
Percentage of respondents using the various apps for personal and professional use.

To evaluate the frequency of use of the apps by the professionals surveyed, we considered the responses in which they had selected the option “often” or “constantly” as an indication of usual use. Among the 483 respondents, 474 (98.1%) were regular users of social networks and 362 (74.9%) also used them in work-related situations. WhatsApp was the most used app, in both personal and professional contexts. Respondents indicated using WhatsApp in 467 of 483 (92.6%) cases and Facebook in 209 (41.5%; Figure 2).

Of the 483 participants, 362 used their mobile phone to access SNSs in a work-related context (74.9%, 95% CI 71.1%-78.8%). This proportion was significantly higher in 3 situations: in the age span between 20 and 30 years (37/44, 84.1%, 95% CI 73.3%-94.9%); among professionals who used their mobile phone more than 3 hours daily (100/118, 84.7%, 95% CI 78.3%-91.2%); and among those with less than 15 years of work experience (142/175, 81.1%, 95% CI 75.3%-86.9%; Table 1).

**Table 1:** Demographic data according to the use of social networking site apps.

Characteristics		Total (N=483)	Users (n=362)	Nonusers (n=121)	Crude odds ratio (95% CI)	*P* value
**Age (years)^a^, n (%)**						
	>50	161	110 (68.3)	51 (31.7)	1	
	41-50	158	120 (75.9)	38 (24.1)	1.46 (0.9-2.4)	.13
	31-40	120	95 (79.2)	25 (20.8)	1.76 (1-3.1)	.04
	20-30	44	37 (84.1)	7 (15.9)	2.45 (1-5.9)	.04
	Median (SD)		44 (10.4)	48 (10.1)		.005^b^
**Sex, n (%)**						
	Male	90	64 (71.1)	26 (28.9)	1	
	Female	393	298 (75.8)	95 (24.2)	1.3 (0.8-2.1)	.35
**Health profession, n (%)**						
	Nurse	211	159 (75.4)	52 (24.6)	1	
	Physician	215	162 (75.3)	53 (24.7)	1 (0.6-1.6)	.99
	Midwife	31	24 (77.4)	7 (22.6)	1.1 (0.5-2.8)	.80
	Social worker	21	14 (66.7)	7 (33.3)	0.5 (0.8-3)	.44
	Dentist	5	3 (60)	2 (40)	0.7 (0.3-1.7)	.38
**Role, n (%)**						
	Clinical	406	301 (74.1)	105 (25.9)	1	
	Clinical and academic	77	61 (79.2)	16 (20.8)	1.3 (0.7-2.4)	.34
**Mobile phone daily use (hours), n (%)**						
	<1	190	126 (66.3)	64 (33.7)	1	
	1-3	175	136 (77.7)	39 (22.3)	1.8 (1.1-2.8)	.01
	>3	118	100 (84.7)	18 (15.3)	2.8 (1.6-5)	.001
**Work experience (years), n (%)^b^**						
	>35	46	29 (63)	17 (37)	1	
	26-35	103	72 (69.9)	31 (30.1)	1.4 (0.7-2.8)	.40
	16-25	159	119 (74.8)	40 (25.2)	1.7 (0.9-3.5)	.11
	≤15 years	175	142 (81.1)	33 (18.9)	2.5 (1.2-5.1)	.01
	Median (SD)	N/A^c^	18 (10.6)	22 (11.3)	N/A	.002^b^

^a^Pearson correlation coefficient (age and work experience) =.9; *P*<.001.

^b^*P* value for the linear trend test (analysis of variance).

^c^N/A: not applicable.

The factors independently associated with the use of apps to access SNSs in a professional-related context were having less than 15 years of work experience (adjusted OR 2.11, 95% CI 1.02-4.36) and a frequency of mobile phone use greater than 3 hours a day (adjusted OR 1.90, 95% CI 1.07-3.38; Table 2).

Most of the 362 respondents (mean 67.5%, SD 6.1%) considered using mobile phones to access SNSs in a professional context as useful or very useful in all 8 domains studied. Considering valuations rated as “useful” or “very useful” as indicators of usefulness, the best-rated domain was problem-solving skills (276/362, 76.2%, 95% CI 71.8%-80.6%), followed by knowledge about the profession (261/362, 72.1%, 95% CI 67.5%-76.7%) and speed and clinical safety (254/362, 70.2%, 95% CI 65.4%-74.9%; Table 3).

When we compared the apps according to the same domains, we observed that WhatsApp, Facebook, and Twitter were well valued for the acquisition of professional knowledge, creativity and innovation, and critical thinking skills. WhatsApp and Facebook were valued positively for their speed in helping to reach clinical decision, whereas WhatsApp was the only app positively valued for problem solving as well (177/219, 80.8%, 95% CI 75.6%-86.0%; Table 4).

We also asked the respondents to select their main reasons for using the apps. The reasons most frequently cited were communication between professionals and drug or clinical consultations (Table 5). Among the reasons added by professionals, 8 of the 362 (2.2%) respondents reported using SNS apps to send photographs to other professionals and 5 of 362 (1.4%) reported using them to register clinical information.

These preferences varied according to the apps preferred by the health care professionals. However, it is notable that communication with other professionals was reported by 213 of 219 (97.3%, 95% CI 95.1%-99.4%) WhatsApp users (Table 4).

**Table 2:** Multivariate analysis of factors associated with work-related use of social networking site apps by primary care professionals.

Associated factors		Adjusted odds ratio (95% CI)	*P* value
**Work experience (years)**			
	>35	1	N/A^a^
	16-35	1.54 (0.79-2.99)	.19
	≤15	2.11 (1.02-4.36)	.04
**Daily use of mobile phone (hours)**			
	<3	1	N/A
	≥3	1.90 (1.07-3.38)	.02

^a^N/A: not applicable.

**Table 3:** Assessment of the usefulness of social networking sites in the 8 domains analyzed (n=362).

Domains	Rating, n (%)			
	Not useful	Of little use	Useful	Very useful
Problem solving	18 (5.0)	68 (18.8)	202 (55.8)	74 (20.4)
Knowledge about profession	25 (6.9)	76 (21.0)	206 (56.9)	55 (15.2)
Speed and clinical safety	23 (6.4)	85 (23.5)	190 (52.5)	64 (17.7)
Patient care	25 (6.9)	85 (23.5)	199 (55.0)	53 (14.6)
Clinical decisions	20 (5.5)	91 (25.1)	203 (56.1)	48 (13.3)
Clinical skills	33 (9.1)	97 (26.8)	185 (51.1)	47 (13.0)
Creativity and innovation	30 (8.3)	112 (30.9)	171 (47.2)	49 (13.5)
Critical thinking	36 (9.9)	116 (32.0)	170 (47.0)	40 (11.0)

**Table 4:** Assessment of the impact of 4 apps compared according to the 8 domains analyzed.

Main uses of the apps		WhatsApp (n=219)	Facebook (n=22)	Twitter (n=20)	Instagram (n=8)
**Domains, n (%)**					
	Problem solving	177 (80.8)^a^	19 (97.6)	15 (75.0)	6 (75.0)
	Knowledge about profession	167 (76.3)^b^	21 (95.5)^a^	17 (85.0)^c^	8 (100)
	Speed and clinical safety	164 (74.9)^c^	21 (95.5)^d^	19 (95.0)^a^	6 (75.0)
	Patient care	155 (70.8)	19 (86.4)	18 (90.0)^e^	6 (75.0)
	Clinical decisions	166 (75.8)^f^	21 (95.5)^g^	17 (85.0)	8 (100)
	Clinical skills	146 (66.7)	20 (90.9)^d^	17 (85.0)	8 (100)
	Creativity and innovation	147 (67.1)^c^	20 (90.9)^h^	18 (90.0)^g^	6 (75.0)
	Critical thinking	140 (63.9)^i^	21 (95.5)^j^	17 (85.0)	6 (75.0)
**Utility, n (%)**					
	Communication with other professionals	213 (97.3)^j^	21 (95.5)	18 (90.0)	8 (100)
	Pharmacological or clinical consultations	124 (56.6)	14 (63.6)	16 (80.0)^k^	6 (75.0)
	Professional development	72 (32.9)	9 (40.9)	11 (55.0)^l^	4 (50.0)
	Health promotion	60 (27.4)	13 (59.1)^j^	14 (70.0)^j^	2 (25.0)
	Communication with patients	50 (22.8)	5 (22.7)	3 (15.0)	1 (12.5)
	Social networks	50 (22.8)	13 (59.1)^j^	12 (47.5)^j^	4 (50.0)
	Work or research opportunities	44 (20.1)^i^	7 (31.8)	5 (25.0)	3 (37.5)
	Other	9 (4.1)	0 (0.0)	0 (0.0)	0 (0.0)

^a^*P*=.01.

^b^*P*=.03.

^c^*P*=.02.

^d^*P*=.04.

^e^*P*=.006.

^f^*P*=.003.

^g^*P*=.007.

^h^*P*=.001.

^i^*P*=.002.

^j^*P*=.005.

^k^*P*<.001.

^l^*P*=.009.

**Table 5:** Reasons given by the professionals (n=362) for using social networking site apps.

Reasons for using the apps^a^		n (%)	95% CI
Communication with other professionals		338 (93.4)	90.8-95.9
Pharmacological or clinical consultations		204 (56.4)	51.2-61.5
Professional development		106 (29.3)	24.6-34.0
Health promotion		86 (23.8)	19.4-28.2
Communication with patients		72 (19.9)	15.8-24.0
Social networks		71 (19.6)	15.5-23.7
Work or research opportunities		57 (15.7)	12.0-19.5
**Other**		19 (5.2)	2.9-7.6
	Sending images or clinical photos	8 (2.2)	0.7-3.7
	Clinical information record	5 (1.4)	0.2-2.6
	Assistance support tools	3 (0.8)	0-1.8
	Professional email	3 (0.8)	0-1.8

^a^Respondents could choose more than 1 reason.

## Discussion

### Principal Findings

The results of this study indicate that most of the primary health care professionals surveyed were using apps to access SNSs in a professional context and that WhatsApp, Twitter, and Facebook, in this order, were the most used, in both personal and professional contexts [20,29]. In terms of its benefits, WhatsApp was generally perceived as more useful for improving professional knowledge and clinical problem solving [13]. These findings suggest that these apps can be powerful tools to involve health professionals in their professional activities and that they can be used as a model to develop new and more secure apps in the future [21].

The study showed a higher proportion of SNS users among professionals with shorter work experience and, although the univariate analysis didn’t achieve statistical significance, a multivariate analysis demonstrated that age and work experience were significantly correlated variables (linear correlation) and, together with hours of mobile phone use, generated a good response model. New generations of professionals, as expected, made greater use of mobile phones and everything that use entails (eg, participating in social networks or conducting internet searches). The health system should be adapted to this, both ethically (for the sharing of photos and patient data) and in relation to documentary and assisted support. If we were to repeat our study in 15 years’ time, it would show a completely different picture.

Professionals perceived that using these apps had an impact in several domains, the most prominent of these being the apps’ role in improving knowledge and problem solving, as well as their speed and clinical security. When we inquired about applied uses, respondents emphasized the use of apps as a communication tool and, although the amount of data we obtained did not allow for deep analysis, a significant number of professionals claimed to have sent patient images or photographs to other colleagues and a small percentage had sent clinical information. Some studies carried out with mobile phones mentioned that telemedicine offers an opportunity to send photos and video clips, representing a source of clinical support for obtaining a second opinion from other colleagues and experts [30,31]. In an environment of scarce resources, the use of mobile phones for medical communication could be of great value. However, we should not forget that sending health information through apps, such as WhatsApp, can imply a serious risk to patient data safety. Professionals are using SNS tools such as WhatsApp and Facebook commonly to communicate and share clinical information, and this use of social media as a health tool raises ethical issues in part because of the possible inappropriate use of individuals’ personal and sensitive information and the possible breach of data security regulations (such as the European Union’s General Data Protection Regulation). Health institutions must give special attention to advising health professionals about these risks.

The use of SNSs as a means of communicating with patients has been reported as being of little use, probably, according to other studies, due to the lack of legal protection, because their use could be a source of errors or distractions [32], or because of the preference for face-to-face contact with their physicians by a large part of the population [33]. This trend could change in the near future, as pointed out by some studies carried out in places where mobile phones are mostly used, since it can improve patient care and make the use of resources more efficient [29-31,34].

### Limitations

The study had several limitations. A selection bias was caused by the type of convenience sample used (closed cohort). This problem could be solved in future research by expanding the recruitment to self-selected professionals on the internet. Another limitation originated in the low response rate and the bias inherent in using a Web-based survey that those with better technology would be likelier to respond and, therefore, more likely to use apps for professional purposes. There was another important bias in relation to the high percentage of physicians and nurses who responded to the questionnaire, caused in part by the higher number of those professional categories registered as electronic health record users and in part by the low participation rate of other clinical categories included in the study.

Because this was a descriptive study, we were not able to establish a cause-effect relationship.

### Comparison With Prior Work

Our findings are in accordance with those observed in other studies [6,11]. The most popular social and messaging platforms used by health professionals were the same, and they had similar usage patterns in their professional context. The limited use of other more specialized groups of health apps in our study differed from the findings of other studies conducted in populations that used these apps constantly, especially those that are used for direct patient management (eg, medical guidelines and medical calculators) [29,34], which could be explained by poor knowledge of the current market or by technological barriers, especially among certain age segments of users. Although there are more specialized health apps that offer similar communication features and tend to have better safety profiles and certification in the handling of data [21], the lack of information and poor knowledge about them could be preventing their use. This leaves open the possibility that promotion and dissemination of such tools in professionals’ environments could improve their use.

Social networking is a form of social media, and SNS users typically download services that offer social media functionality to their mobile devices (eg, mobile phones and tablet computers), but they can also access SNSs on desktop computers or laptops [35]. Studies to determine which devices health professionals use to access social media are lacking. Some sources suggest that the rate of using mobile phones or mobile devices to using computers and laptops for accessing social media is 2 to 1 [36]. Our study specifically focused on accessing SNSs through mobile device apps, assuming that these devices are used most frequently, but more studies need to be done on this particular subject.

Some studies found that physicians use predetermined browsers in their mobile phones to access SNSs, search clinical practice guidelines or patient information, or access medical information through the Web [37,38]. Our survey may not have caught this functionality, carried out with mobile phones. Other studies reported the use of mobile technology in primary care as a good tool to provide medical care in hard-to-reach areas, making it easier to guarantee health services and resources [11,18,19]. The combination of SNSs and mobile health offers a great opportunity to strengthen information systems transforming health systems. However, the implementation of this combination should carefully consider aspects such as the security, privacy, and confidentiality of user information, but it also needs to take into consideration health professionals’ preferences [20,22,23]. The results of this study provide new insights into the use and perceived benefits of apps among primary care professionals and, specifically, about the uses and needs relating to social networks. The demonstration of health professionals’ use of SNSs should warn us about the need to improve and enhance their benefits, but also to facilitate the proper and secure use of these new tools. Further analytical-experimental research using more exhaustive methods to recruit participants will be essential to confirm and extend the results of this study.

### Conclusions

The vast majority of primary health care professionals surveyed, 362 of 483 (74.9%) respondents, accessed SNSs with their mobile phones in a work-related context. WhatsApp was the most used, in both personal and professional contexts. Mobile phone apps with access to SNSs in health care are frequently used for communication between professionals, but they are also used for the exchange of files and images or recorded clinical data. The use of these apps, according to the professionals surveyed, affects problem solving, but their use for communicating with patients is not yet widespread. We recommend that health institutions assess the need to improve the general and specific knowledge about the available apps and, thereby, improve and facilitate their use among health professionals as a way to prevent the risks of inappropriate use.

## Abbreviations

OR: odds ratio
SNS: social networking site
